# The Effect of Granulocyte Colony-Stimulating Factor on the Progression of Atherosclerosis in Animal Models: A Meta-Analysis

**DOI:** 10.1155/2017/6705363

**Published:** 2017-09-12

**Authors:** Manli Liu, Kejian Liu, Dongdong Chen, Hongzhi Chen, Kunming Sun, Xinxin Ju, Jiaojiao Lan, Yang Zhou, Weishan Wang, Lijuan Pang

**Affiliations:** ^1^Department of Pathology and Key Laboratory of Xinjiang Endemic and Ethnic Diseases, Ministry of Education, Shihezi University School of Medicine, Shihezi, Xinjiang, China; ^2^Department of Pathology, The First Affiliated Hospital to Shihezi University School of Medicine, Shihezi, Xinjiang, China; ^3^Department of Cardiology, The First Affiliated Hospital to Shihezi University School of Medicine, 45 North 3rd Road, Shihezi, Xinjiang, China; ^4^Department of Cardiology, Tongji Hospital, Tongji Medical College, Huazhong University of Science and Technology, 1095 Jiefang Avenue, Wuhan, China; ^5^Department of Orthopaedics, Shihezi University School of Medicine, 45 North 3rd Road, Shihezi, Xinjiang, China

## Abstract

**Background:**

Atherosclerosis is a common inflammatory disease. Stem cell and endothelial progenitor cell treatments can improve cardiac function after myocardial infarction. Granulocyte colony-stimulating factor (G-CSF) is a mobilisation agent, mobilising stem cells from the bone marrow to circulation in the blood. G-CSF may constitute a treatment of atherosclerosis. We have conducted meta-analysis to evaluate the current evidence for the effect of G-CSF on the progression of atherosclerosis in animal models and to provide reference for preclinical experiments and future human clinical trials of atherosclerosis treatment.

**Methods:**

We searched several databases and conducted a meta-analysis across seven articles using a random-effect model. All statistical analyses were performed using Review Manager Version 5.2 and Stata 12.0.

**Results:**

We found that G-CSF therapy was associated with reduced atherosclerotic lesion area (weighted mean difference (WMD): 7.29%; 95% confidence interval (CI): 2.06–12.52%; *P* = 0.006). No significant differences in total serum cholesterol (*P* = 0.54) and triglyceride levels (*P* = 0.95) were noted in G-CSF treatment groups compared with controls. Multivariable metaregression analysis revealed that the animal type (rabbit, *P* = 0.022) and frequency of G-CSF administration (>20, *P* = 0.007) impacted the atherosclerotic lesion area changes.

**Conclusion:**

The meta-analysis suggested that G-CSF treatment might inhibit the progression of atherosclerosis in animal models.

## 1. Introduction

Atherosclerosis is a common disease with serious implications for the human health. It is the main pathological basis of ischemic cardiovascular disease, including coronary heart disease, cerebrovascular disease, and thromboembolic disease [1]. Atherosclerosis begins with an impairment of vascular endothelial function and structure [2–4].

Granulocyte colony-stimulating factor (G-CSF), a prototypical mobilising agent, induces hematopoietic stem/progenitor cell mobilisation [5, 6] and stimulates angiogenesis-related endothelial cell proliferation and migration [7]. In the clinic, the source of allogeneic stem cells for transplantation treatment of aplastic anaemia and other diseases usually comprised G-CSF-mobilised peripheral blood stem cells [8]. Stem cells and endothelial progenitor cells promote angiogenesis and repair endothelial injury [9, 10]. Moreover, some studies suggest that stem cell and endothelial progenitor cell treatment can reduce atherosclerosis plaque [11–13]. Guo et al.' study showed that G-CSF treatment was beneficial in treating acute myocardial infarction [14, 15]. In addition, Arai et al. [16] reported that the effect of G-CSF therapy in the treatment of atherosclerotic peripheral arterial disease was the same as that of bone marrow transplantation.

Animal models comprise a valuable tool for preclinical studies, informing the development of treatment approaches for human diseases. A number of animal experiments have been carried out to study the effect of G-CSF on the progression of atherosclerosis. However, the potential of G-CSF treatment remains controversial. The basic function of G-CSF is to stimulate the proliferation of neutrophil progenitor cells [17]; furthermore, neutrophils may be a risk factor for atherosclerosis and progression of atherosclerosis [18]. While several studies showed that G-CSF was applicable for the treatment of atherosclerosis [19–24], some reached different or even opposite conclusions [20, 25]. Consequently, in this study, we conducted a meta-analysis of the published animal model data to assess the effect of G-CSF on the progression of atherosclerosis.

## 2. Materials and Methods

### 2.1. Search Strategy

We searched the following databases: PubMed (https://www.ncbi.nlm.nih.gov/pubmed), EMBASE (https://www.elsevier.com), the Chinese BioMedical Literature (CBM, http://www.sinomed.ac.cn/), and China National Knowledge Infrastructure (CNKI, http://www.cnki.net/), up to July 4, 2016. The following keywords and their combinations were used: “Granulocyte colony-stimulating factor or G-CSF” and “atheroscleroses or atherogenesis or atherosclerosis”.

### 2.2. Inclusion and Exclusion Criteria

Two reviewers (MLL and LJP) independently qualified all studies. Qualified studies had to satisfy the following experimental criteria: (1) randomised controlled trial of an arterial atherosclerosis animal model; (2) animal model without vascular injury or artery narrowing rings; (3) injection G-CSF as the only experimental intervention measure; and (4) evaluation of the degree of atherosclerosis (atherosclerotic lesion area ratio, total serum cholesterol, and triglyceride levels) as the final results. Principal criteria for the exclusion of studies were as follows: (1) irrelevant topic, duplicate, review, discussion, and comment; (2) no appropriate data; and (3) data partly published in another journal.

### 2.3. Data Extraction

All data from the qualified studies were independently extracted by the two reviewers. The following data types were extracted: basic characteristics of the animal model atherosclerotic lesion area, total serum cholesterol levels, total serum triglyceride levels, G-CSF dose, total number of injections, and the time point of atherosclerotic lesion area ratio measurements. If required, data were estimated from graphic elements provided in the qualified studies [26].

### 2.4. Statistical Analysis

Our main indicator of treatment success was the difference of mean atherosclerotic lesion areas between experimental and control groups. We analysed the data using a random-effect model, but heterogeneity test revealed significant heterogeneity (*P* < 0.1). Multivariable metaregression analysis was used to determine the factors that underpin the heterogeneity, followed by subgroup analysis. Multivariable metaregression analysis was performed with the following factors that could lead to heterogeneity: animal type (rabbits, mice); route of delivery (hypodermic, intravenous, or intraperitoneal injection); G-CSF dose (≤100 *μ*g/kg/d or >100 *μ*g/kg/d); the total number of injections (≤20 or >20); the time point of atherosclerotic lesion area measurement (from the start of treatment: ≤6 weeks, 8 or 9 weeks, and 12 weeks). Subgroup analyses were performed for the same factors as above. Continuous variables were expressed as weighted mean differences (WMDs) with 95% confidence intervals (CIs) between the control groups and G-CSF-treated animals. *P* < 0.05 was deemed statistical significant.

We used the funnel plot to assess the publication bias. All statistical analyses were performed using Review Manager Version 5.2 (Copenhagen: The Nordic Cochrane Centre, The Cochrane Collaboration, 2012) and Stata 12.0.

## 3. Results

### 3.1. Characteristics of Eligible Studies

**Figure 1:**
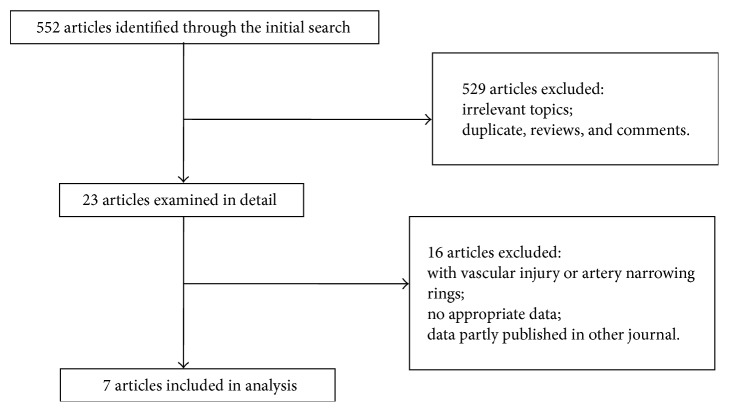
Flow diagram of the meta-analysis.

Articles in English (496) and in Chinese (56) were retrieved for this study from databases specified in Section 2.1. (Figure 1). Following the screening, seven articles met our inclusion criteria [19–25]. The details of the included studies are presented in Table 1. Four articles used mouse animal model and three articles used rabbit.

### 3.2. Results of Statistical Analyses

**Figure 2:**
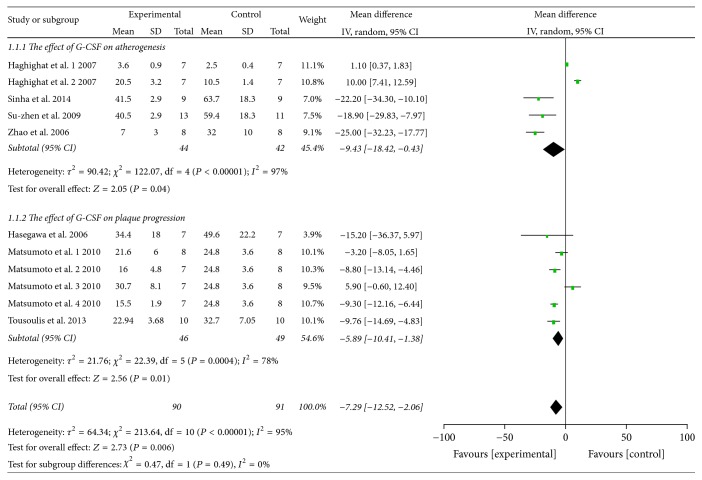
Forest plot showing the effect of G-CSF therapy on atherosclerotic lesion area (%), compared with controls. IV, independent variable; 95% CI, 95% confidence interval.

**Figure 3:**
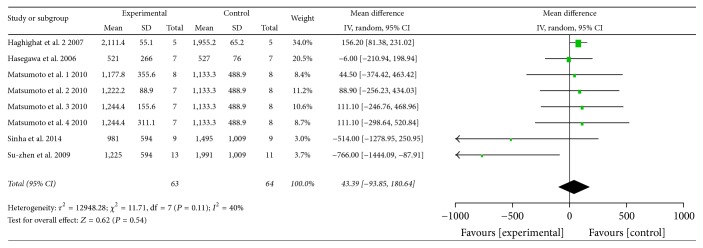
Forest plot showing the effect of G-CSF treatment on total cholesterol levels (mg/dl), compared with controls. IV, independent variable; 95% CI, 95% confidence interval.

**Figure 4:**
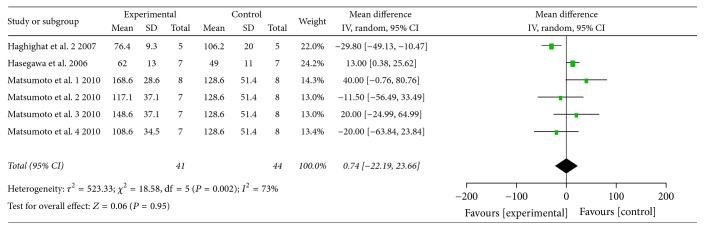
Forest plot showing the effect of G-CSF treatment on triglyceride levels (mg/dl), compared with controls. IV, independent variable; 95% CI, 95% confidence interval.

Pooled analysis revealed that the atherosclerotic lesion area was 7.29% after G-CSF injection and was significantly lower than in the control group (95% CI: 2.06–12.52%;* Z* = 2.73; *P* = 0.006), with significant heterogeneity (*P* < 0.00001, *I*^2^ = 95%; Figure 2). When treatment is before or at the same time as high-fat diet commencement, the atherosclerotic lesion area was 9.43% lower in the G-CSF group than in the control group (95% CI: 0.43–18.42%;* Z* = 2.05; *P* = 0.04; Figure 2). Also in case of treatment in animals with existing atherosclerosis, the atherosclerotic lesion area was 5.89% lower in the G-CSF group than in the control group (95% CI: 1.38–10.41%;* Z* = 2.56; *P* = 0.01; Figure 2). The total serum cholesterol levels were not significantly different between the experimental and control groups (WMD: 43.39 mg/dl; 95% CI: −93.85–180.64 mg/dl;* Z* = 0.62; *P* = 0.54; Figure 3). Similarly, triglyceride levels were not significantly different between the experimental and control groups (WMD: 0.74 mg/dl; 95% CI: −22.19–23.66 mg/dl;* Z* = 0.06; *P* = 0.95; Figure 4). Multivariable metaregression analysis revealed that the types of animals (rabbit, *P* = 0.022) and the frequency of G-CSF administration (>20, *P* = 0.007) were significantly associated with the reduction in atherosclerotic lesion area.

**Figure 5:**
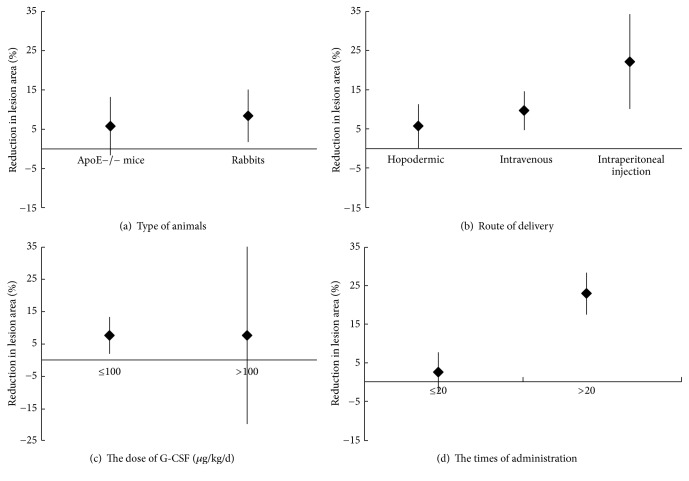
Subgroup analysis showing a trend towards greater reduction in atherosclerotic lesion area (%) after G-CSF treatment compared with controls, considering (a) animal type (*P* = 0.022); (b) delivery route (*P* = 0.133); (c) G-CSF dose (≤100 *μ*g/kg/d; *P* = 0.080); (d) total number of injections (>20, *P* = 0.007).

The results of subgroup analyses are shown in Figure 5. In studies with rabbit models of atherosclerosis, the atherosclerotic lesion area ratio (95% CI: 1.75–15.10%; *P* = 0.01) was 8.42% lower in the G-CSF treatment group than in the control group. In mouse model studies, the atherosclerotic lesion area was 5.78% (95% CI: −1.64–13.21%) smaller in the G-CSF group relative to the control group. In studies using intraperitoneal G-CSF injection (atherosclerotic lesion area, WMD: 22.20%; 95% CI: 10.10–34.30%) and intravenous injection (atherosclerotic lesion area WMD: 9.76%; 95% CI: 4.83–14.69%), the lesion areas were lower compared with a hypodermic injection treatment (WMD: 5.68%; 95% CI: 0.07–11.29%). After administration of high doses of G-CSF (>100 *μ*g/kg/d), the atherosclerotic lesion area was 7.67% smaller in the G-CSF group than in the matched control group (95% CI: −19.85–35.19%). After administration of low doses of G-CSF (≤100 *μ*g/kg/d), the atherosclerotic lesion area was significantly smaller (WMD: 7.72%; 95% CI: 1.96–13.48%; *P* = 0.009) than in controls. Multiple injections of G-CSF (>20) resulted in a greater reduction of the atherosclerotic lesion area (WMD: 22.95%; 95% CI: 17.55–28.35%; *P* < 0.00001) in the experimental group compared with a matched control group. The atherosclerotic lesion area after ≤20 G-CSF injections was 2.55% (95% CI: −2.56–7.66%) smaller in the experimental group than in a matched control group.

The atherosclerotic lesion area ratio measured >12 weeks since the commencement of G-CSF treatment was significantly lower in the G-CSF group compared with a matched control group (WMD: 7.94%; 95% CI: 0.94–14.94%; *P* = 0.03). The atherosclerotic lesion area measured at 8 or 9 weeks (WMD, 4.61%; 95% CI: −3.69–12.91%) and ≤6 weeks since the beginning of treatment (WMD: 10.04%; 95% CI: 5.24–14.84%) was lower in the experimental group compared with matched controls.

## 4. Discussion

The reported meta-analysis was based on seven rigorously selected published animal model studies to determine the effect of G-CSF on the progression of atherosclerosis. The meta-analysis revealed that G-CSF might inhibit the progression of atherosclerosis, including atherosclerosis and plaque progression, as evidenced by significantly reduced atherosclerotic area after G-CSF injection. Multivariable metaregression analysis revealed that the type of animals and the total number of injections were significantly associated with a reduction in atherosclerotic lesion area ratio. In addition, our subgroup analysis indicated a greater decrease in atherosclerotic lesion area with nonsubcutaneous G-CSF injection but the results were not affected by G-CSF doses and the time when the areas of atherosclerotic lesion were measured (since the initiation of treatment).

G-CSF regulates proliferation, survival, and differentiation of hematopoietic stem cells/hematopoietic progenitor cells. It is one of the hematopoietic growth factor family members [27]. Previous studies suggested that stem cells or endothelial progenitor cells can alleviate atherosclerosis in animal models [11, 13]. G-CSF is recognised as a mobilisation agent of bone marrow stem cells [28] and it has been proposed that G-CSF could comprise one option for the treatment of atherosclerosis. Compared with stem cell transplantation, G-CSF treatment may be more convenient, circumventing bone marrow aspiration [29].

Atherosclerosis is a type of inflammatory disease [2]. Atherosclerotic plaque rupture or erosion may eventually lead to acute myocardial infarction or stroke [30]. Many meta-analyses address the role of G-CSF in the treatment of acute myocardial infarction in patients. These analyses suggest that G-CSF treatment is safe and has minor side effects [29, 31–35]. However, G-CSF treatment has not been introduced into the clinic because some animal studies on G-CSF application in atherosclerosis yielded different results and this lack of effect in clinical trials [19–25]. Some studies had shown that G-CSF helped to reduce the atherosclerotic plaque area [19–24], while Haghighat et al. [25] suggested that it led to increased atherosclerotic lesion area [20, 25].

In the present meta-analysis, the atherosclerotic lesion area ratios, total cholesterol, and triglyceride levels were analysed. G-CSF might inhibit the atherosclerotic process since it can effectively reduce the area of atherosclerotic lesions. However, no significant differences were reported for the total cholesterol and triglyceride levels between the experimental and control groups. We hypothesized that the effect of G-CSF on the size of atherosclerotic lesions might be associated with its ability to mobilise stem cells rather than with its effect on the total serum levels of the above compounds. Tousoulis et al. [23] showed that the area of atherosclerotic lesion in atherosclerotic mice was significantly reduced compared with the control group when they were injected with bone marrow derived progenitor cells (lin−/sca-1+ cells) or endothelial progenitor cells and, in their preliminary experiments, the number of sca-1+/c-kit+/lin− cells was increased after G-CSF treatment. Similarly, Zhao et al.'s [24] data showed that G-CSF increased the number of endothelial progenitor cells and decreased plaque area compared to control. In other published research, the meta-analysis of Zohlnhöfer et al. confirmed that G-CSF could mobilise CD34+ stem cells from bone marrow to peripheral blood in a dose-dependent manner [35]. Rauscher et al. [36] suggested that bone marrow derived progenitor cells from young nonatherosclerotic mice can prevent the progression of atherosclerosis in ApoE−/− mice; they thought it was because progenitor cells can replace aging endothelial cells to repair the blood vessels and fight atherosclerosis. Thus, G-CSF may reduce the area of atherosclerotic lesions by mobilising progenitor cells.

G-CSF had been shown to increase the number of neutrophils in the circulation by promoting the differentiation of bone marrow progenitor cells into neutrophils and accelerating the maturation and release rate of neutrophils [37, 38]. G-CSF regulated neutrophil apoptosis by maintaining the levels of antiapoptotic MCL-1 and inhibiting the expression of proapoptotic Bcl-2 family member Bax [39]. Moreover, G-CSF was found to increase the adhesion and phagocytosis of mature neutrophils [40, 41]. Drechsler et al.'s study on ApoE−/− mice showed a positive correlation between plaque size and the number of neutrophils in early atherosclerosis [42]. In the study of human arteries, it was found that neutrophil degranulation marker levels were higher in culprit-stenosing plaques containing intraplaque hemorrhages than other [43], and there was a positive correlation between the high blood vessel density and the number of neutrophils [44]. These showed that the increase of neutrophils was closely related to atherogenesis, atherosclerosis progression, and driven plaque rupture [18, 45]. In the articles included in our meta-analysis, Sinha et al. [21] considered that neutrophils were increased after G-CSF treatment compared with the control group, but with no statistical significance. Matsumoto et al. [20] found that in case of G-CSF injection for 5 days, the number of neutrophils was increased within 6 days after the treatment and gradually decreased after 6 days. Interestingly, their final results have shown that G-CSF treatment reduced the atherosclerosis area. It may be that the potential benefits of G-CSF outweigh these hazards. The detailed mechanism of the effects of G-CSF on atherosclerosis remains to be further studied.

The effect of G-CSF on atherosclerotic plaque stability remains unresolved. Plaque vulnerability was characterized by a large necrotic lipid core, thin fibrous cap, and high content of macrophage [46]. In the study by Sinha et al. [21] and Su-zhen et al. [22], G-CSF reduced the infiltration of lipids and macrophages into the atherosclerotic lesions. Matsumoto et al.'s [20] study indicated that G-CSF increased the accumulation of collagenous fibers and elastin in plaque. MMPs were believed to contribute to plaque rupture, thinning the fibrous cap by degrading extracellular matrix and collagen [46]. Matsumoto et al. [20] also found that G-CSF increased the expression of matrix metalloproteinase inhibitor TIMP-2 mRNA in aorta. These results suggested that G-CSF can increase the stability of plaque. However, Sinha et al. [21] and Su-zhen et al. [22] found that G-CSF increased the expression of MT1-MMP. High expression of MT1-MMP can promote plaque damage [47]. Therefore, the effect of G-CSF on plaque stability requires additional experimental investigation in the future.

Furthermore, our findings revealed that the effect of G-CSF treatment on atherosclerosis progression was significantly affected by the type of animal model and the frequency of G-CSF administration, with rabbit models and multiple administration (>20) resulting in a more pronounced decrease of atherosclerotic lesion area ratio. It is likely that the effect of G-CSF on large blood vessels (rabbit) is greater than on small blood vessel (mouse). This result may also be related to the difference between species. The results of subgroup analyses indicated that the effect of low-dose G-CSF (≤100 *μ*g/kg/d) treatment on atherosclerosis progression was not different from high-dose G-CSF treatment (>100 *μ*g/kg/d). But clinical data revealed that low-dose G-CSF treatment is safe in patients with stroke or acute myocardial infarction (<10 *μ*g/kg/d) [48, 49] and the maximum safe dose of G-CSF was determined as 100 *μ*g/kg/d in patients with acute ischemic stroke [50]. In addition, intravenous and intraperitoneal G-CSF injections were more effective than subcutaneous injections. Moreover, the assessment of the effect of G-CSF on atherosclerosis progression was not significantly affected by the time since therapy commencement when the area of atherosclerotic lesions was measured. However, the analysed studies may not have been long enough to observe all consequences of G-CSF treatment, with the longest study period of 12 weeks [20, 24]. Therefore, long-term efficacy of G-CSF treatment remains unclear. Overall, these results suggest that future evaluation of G-CSF as a treatment for atherosclerosis should focus on multiple G-CSF. Future preclinical experiments need to address the effects of G-CSF on atherosclerosis in different animal models, with different routes of administration and for longer treatment periods.

The Reviewing Animal Trials Systematically (RATS) Group [51] suggested that the outcomes of many studies involving animal models of disease pertaining to the potential treatment of human were not utilised because they were poorly conducted and evaluated without systematic reviews. At the same time, the best approach for demonstrating clinical significance of animal experiments comprises conducting systematic overview and, if possible, comparing the conclusions with the outcomes of corresponding clinical trials [51]. We here conducted such a systematic review and meta-analysis of animal model data to evaluate the therapeutic effect of G-CSF in atherosclerosis. Our results verified that G-CSF treatment might indeed inhibit the progression of atherosclerosis when an appropriate dose and course of G-CSF administration are selected. We suggest that evaluating the efficacy and safety of G-CSF in atherosclerosis treatment in large animal experiments and human clinical trials is required before clinical use of G-CSF.

## 5. Conclusions

We here adopted multiple regression analysis to evaluate the effect of various factors on the progression of atherosclerosis in animal models. We discovered that the type of animal model and the frequency of G-CSF administration impact the G-CSF treatment efficacy. Keeping in mind that the analysis was based on published data rather than on accessed individual data, our meta-analysis effectively summarises the available data from animal experiments, drawing conclusions about the efficacy of G-CSF therapy and providing a reference for future clinical treatment of atherosclerosis.

## Figures and Tables

**Table 1:** Characteristics of the studies.

First author (year)	*n*	Diet	Animal model	Delivery route	G-CSF^a^ dose	Total number of injections	Time point of atherosclerotic lesion area measurement^b^
Haghighat (2007)	7 7	Low-fat Atherogenic	Mouse Mouse	Hypodermic	10 *μ*g/kg/d 10 *μ*g/kg/d	20 20	8 weeks 8 weeks

Hasegawa (2006)	7	Not reported	Rabbit	Hypodermic	100 *μ*g/kg/d	7	4 weeks

Matsumoto (2010)	7 Or 8	Atherogenic	Rabbit	Hypodermic	50 *μ*g/kg/d 100 *μ*g/kg/d 300 *μ*g/kg/d 100 *μ*g/kg/d	5 5 5 15	12 weeks 12 weeks 12 weeks 12 weeks

Sinha (2014)	9	Atherogenic	Mouse	Intraperitoneal injection	300 *μ*g/kg/d	45	9 weeks

Su (2009)	13 or 11	Atherogenic	Mouse	Hypodermic	10 *μ*g/animal/d	63	9 weeks

Tousoulis (2013)	10	Atherogenic	Mouse	Intravenous	100 *μ*g/kg/d	7	6 weeks

Uchiyama (2012)	10	Atherogenic	Mouse	Hypodermic	200 *μ*g/kg/d	20	4 weeks

Zhao (2006)	8	Atherogenic	Rabbit	Hypodermic	50 *μ*g/animal/d	84	12 weeks

^a^G-CSF, granulocyte colony-stimulating factor. ^b^Time from the beginning of treatment.
